# Heightened orofacial, manual, and gait variability in Parkinson’s disease results from a general rhythmic impairment

**DOI:** 10.1038/s41531-019-0092-6

**Published:** 2019-09-25

**Authors:** Frédéric Puyjarinet, Valentin Bégel, Christian Gény, Valérie Driss, Marie-Charlotte Cuartero, Sonja A. Kotz, Serge Pinto, Simone Dalla Bella

**Affiliations:** 1 EuroMov Laboratory, University of Montpellier, 700 Avenue du Pic Saint Loup, 34090 Montpellier, France; 2 Charles-de-Gaulle University, Lille 3, 42 Rue Paul Duez, 59 000 Lille, France; 3 Neurology Department, CHRU of Montpellier, 80 Avenue Augustin Fliche, 34000 Montpellier, France; 4 Investigation Clinic Center, CHRU of Montpellier, 80 Avenue Augustin Fliche, 34000 Montpellier, France; 5 Aix Marseille Univ, CNRS, LPL, Aix-en-Provence, France; 6 Department of Neuropsychology and Psychopharmacology, University of Maastricht, Universiteitssingel, 6200 MD Maastricht, Netherlands; 7 International Laboratory for Brain, Music and Sound Research (BRAMS), 90 Vincent-d’Indy Ave., Outremont, QC H2V 2S9 Canada; 8 Department of Psychology, University of Montreal, 2900 Boulevard Edouard-Montpetit, Montréal, QCH3T 1J4 Montreal, Canada; 9 University of Economics and Human Sciences in Warsaw, Okopowa59, 01-043 Warsaw, Poland

**Keywords:** Neurological manifestations, Parkinson's disease

## Abstract

Individuals with Parkinson’s disease (PD) experience rhythm disorders in a number of motor tasks, such as (i) oral diadochokinesis, (ii) finger tapping, and (iii) gait. These common motor deficits may be signs of “general dysrhythmia”, a central disorder spanning across effectors and tasks, and potentially sharing the same neural substrate. However, to date, little is known about the relationship between rhythm impairments across domains and effectors. To test this hypothesis, we assessed whether rhythmic disturbances in three different domains (i.e., orofacial, manual, and gait) can be related in PD. Moreover, we investigated whether rhythmic motor performance across these domains can be predicted by rhythm perception, a measure of central rhythmic processing not confounded with motor output. Twenty-two PD patients (mean age: 69.5 ± 5.44) participated in the study. They underwent neurological and neuropsychological assessments, and they performed three rhythmic motor tasks. For oral diadochokinesia, participants had to repeatedly produce a trisyllable pseudoword. For gait, they walked along a computerized walkway. For the manual task, patients had to repeatedly produce finger taps. The first two rhythmic motor tasks were unpaced, and the manual tapping task was performed both without a pacing stimulus and musically paced. Rhythm perception was also tested. We observed that rhythmic variability of motor performances (inter-syllable, inter-tap, and inter-stride time error) was related between the three functions. Moreover, rhythmic performance was predicted by rhythm perception abilities, as demonstrated with a logistic regression model. Hence, rhythm impairments in different motor domains are found to be related in PD and may be underpinned by a common impaired central rhythm mechanism, revealed by a deficit in rhythm perception. These results may provide a novel perspective on how interpret the effects of rhythm-based interventions in PD, within and across motor domains.

## Introduction

Among the symptoms of idiopathic Parkinson’s disease (PD), patients often experience a poor appraisal of rhythmic events. When patients are asked to move spontaneously or to the beat of an auditory stimulus (e.g., a metronome or music), timing deficits are found consistently in tasks such as manual tapping.^1^ These rhythmic abilities are known to engage subcortico-cortical networks involving the basal ganglia and the cerebellum,^1–3^ some of which are affected in the progression of the disease. Rhythmic disorders in PD are found in other motor abilities, such as orofacial rhythmic coordination (e.g., oral diadochokinesis tasks), where patients have difficulties in keeping a steady—isochronous—oral rhythm,^4^ or gait, typically showing altered stride timing.^5^ Rhythm disorders in PD manifest also in perceptual tasks, in the absence of motor output, such as extracting the beat from a musical sequence.^6,7^ Altogether, these findings point towards a general rhythm disorder, so-called “general dysrhythmia”, putatively linked to the malfunctioning of a neural circuitry devoted to rhythm processing, which might characterize PD and manifest across different effectors.^8^

This hypothesis entails that rhythmic impairments across these domains should be correlated. Moreover, poor performance across rhythmic motor tasks should be accounted for by rhythmic non-motor tasks. Indeed performing a beat perception task recruits similar subcortico-cortical rhythm structures^6^ as motor tasks. Evidence is scant on the relations between the three aforementioned motor domains and to date, studies have investigated only one or two effectors^8^ at the same time in the same patients. Moreover, in none of these studies was rhythm assessed in a non-motor task. The above-mentioned hypothesis of general dysrhythmia is appealing as it would provide a parsimonious account of a number of rhythm disorders in PD. In fact, regarding rhythm-based management interventions, beneficial effects of training rhythmic skills in a given motor domain (e.g., gait) may theoretically and ideally transfer to other domains (e.g., oral articulation). Thus, the goal of this study is to fill this gap, to test the possibility that rhythm disorders in various motor domains in PD may derive from a common impaired central mechanism for rhythm processing. To this aim: (1) we examined relations between rhythm skills in oral diadochokinesia, finger tapping, and gait tasks and (2) we tested whether rhythm perception could predict rhythmic performance across these three motor domains.

## Results

### Relationships between rhythmic motor domains

**Fig. 1:**
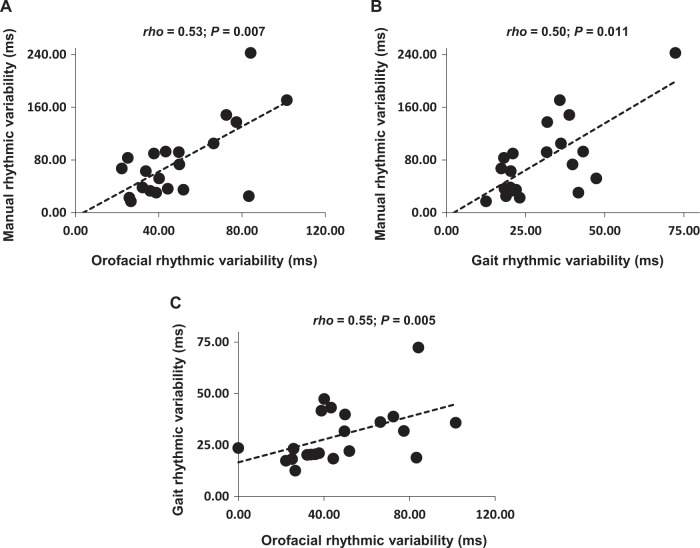
Pair-wise correlations of rhythmic variability in the three motor tasks. Excluding extreme scores that were apparent in manual and gait domains did not alter the results. Manual rhythmic variability refers to the paced condition. Correlation tests were pair-wise Spearman’s rank correlations, *rho*

Results obtained in rhythm production and rhythm perception tasks are summarized in Table 1. High manual rhythmic variability in the paced task was linked to high variability in both orofacial (*rho* = 0.53; *P* = 0.007) and gait (*rho* = 0.50; *P* = 0.011) domains. In turn gait variability was linked to orofacial variability (*rho* = 0.55; *P* = 0.005). Correlations are reported in Fig. 1. Rhythmic variability in the unpaced manual task was correlated with gait variability (*rho* = 0.44; *P* = 0.02), but not significantly to orofacial variability (*rho* = 0.18; *P* = 0.20).

**Table 1:** Results obtained in the oral diadochokinesia, finger tapping, and gait tasks

Domain of motor rhythm	Mean (SD)	*n*
Orofacial		
IVI (ms)	183.90 (34.07)	22
IVIs SD	48.68 (22.50)	22
Manual		
Paced		
ITI (ms)	481.43 (121.72)	21
ITIs SD	78.87 (57.25)	21
Unpaced		
ITI (ms)	585.80 (289.80)	22
ITIs SD	147.20 (185.65)	22
Gait		
STI (ms)	1112.65 (114.26)	22
STIs SD	29.75 (13.86)	22
Rhythm perception		
* d*′	1.91 (0.90)	21

Note that we could not obtain the rhythm perception and the finger tapping scores for one patient due to issues in data recording

*IVI* inter-vowel interval, *ITI* inter-tap interval, *SD* standard deviation, *STI* stride time interval

Only a few significant correlations were found between rhythmic variability (Table 1), demographic, clinical and cognitive characteristics (Table 2); severe motor symptoms appearing from the motor examination (part III) of the MDS-UPDRS total score were linked with high gait (*rho* = 0.69, *P* < 0.001) and paced manual (*rho* = 0.45, *P* = 0.044) variability, but not significantly to unpaced manual variability (*rho* = 0.35, *P* = 0.110) or orofacial variability (*rho* = 0.39, *P* = 0.073). Notably, orofacial, unpaced manual, and gait rhythmic variabilities were not significantly related to age and disease duration, Hoehn and Yahr stage, or any measure of cognitive functioning.

**Table 2:** Demographic and clinical characteristics of the PD patients.

	Mean (SD)	*n*
Demographic		
Age (years)	69.50 (5.45)	22
Females	–	5
Males	–	17
Disease duration (years)	11.10 (6.04)	22
Age at onset	58.24 (8.08)	22
Clinical characteristics		
MDS-UPDRS		
Total score	60.29 (19.71)	21
Motor subscore (part III)	31.71 (10.03)	21
Speech item (3.1)	1.43 (0.97)	21
Finger tapping item (3.4)	2.47 (1.63)	21
Gait item (3.10)	0.76 (0.62)	21
Hoehn and Yahr score	2.28 (0.46)	22
Minibest Test	22.05 (4.44)	21
Neuropsychological assessment		
MoCA	25.81 (2.38)	21
Stroop test		
Naming interference time	111.50 (77.72)	20
Naming interference errors	5.30 (6.10)	20
Trail making test		
B/A ratio	2.91 (1.14)	21
Digit Span (forward)	8.90 (1.94)	21
Digit Span (backward)	5.76 (1.73)	21

Maximal scores are 4 for speech item (3.1), 8 for finger tapping item (3.4), and 4 for gait item (3.10) of the MDS-UPDRS. Due to fatigue, note that some patients were not able to undergo the whole set of clinical or neuropsychological examinations

*MDS-UPDRS* Movement Disorder Society—Unified Parkinson’s Disease Rating Scale, *MoCA* Montreal Cognitive Assessment

To test whether variability in the three motor domains can be explained by performance in rhythm perception we used logistic regression. To this aim, we distinguished the less variable patients from the more variable ones. In each rhythmic production domain, the two subgroups differed in terms of variability (Table 3).

**Table 3:** Comparison between the two PD subgroups (the less variable versus the more variable PD patients), and corresponding descriptive statistics

	Less variable		More variable		*W*	*P*
	Mean (SD)	*n*	Mean (SD)	*n*		
Orofacial	31.52 (6.19)	11	65.84 (19.43)	11	0.00	<0.001
Manual						
Paced	35.32 (13.68)	10	118.50 (52.60)	11	0.00	<0.001
Unpaced	34.31 (13.29)	11	260.10 (210.98)	11	0.00	<0.001
Gait	19.30 (2.84)	11	40.20 (12.46)	11	0.00	<0.001

Note that we could not obtain the paced finger tapping score (manual domain) from one patient due to issues in data recording. Comparison tests were Mann–Whitney *U*-tests

### Rhythm perception skills and rhythmic motor domains

**Fig. 2:**
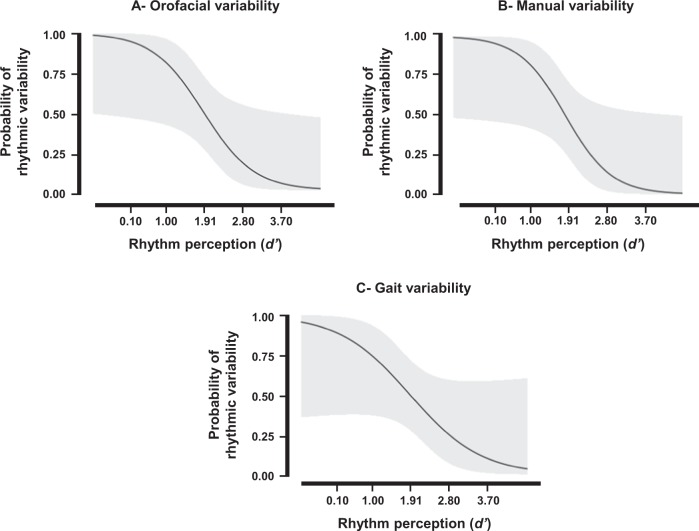
Probability curves of rhythmic variability for logistic regression models. Regression models relative to the oral diadochokinesia **a**, paced finger tapping **b**, and gait **c** tasks. The *y*-axis provides the probability for patients to display high or low rhythmic variability in each motor domain (0 = lowest variability; 1 = highest variability). The *x*-axis corresponds to the rhythm perception performance (*d*′ values). Values in bold indicate the mean ± 1SD values of rhythm perception. Light gray areas refer to the 95% confidence intervals

Logistic regression models showed that perceptual rhythmic skills (i.e., non-motor rhythmic skills, indicated by the sensitivity index, *d*′, in a perception task) predicted rhythmic variability in the three motor domains (Fig. 2): (i) oral diadochokinesia (*P* = 0.013; *χ*^2^ = 6.192; Nagelkerke *R*^2^ = 0.34; AIC = 26.87), (ii) paced finger tapping (*P* = 0.010; *χ*^2^ = 6.701; Nagelkerke *R*^2^ = 0.38; AIC = 25.02), and (iii) gait (*P* = 0.043; *χ*^2^ = 4.112; Nagelkerke *R*^2^ = 0.23; AIC = 28.95). In contrast, perceptual rhythmic skills did not predict rhythmic variability in the unpaced manual task (*P* = 0.975). These results remained unchanged when controlling for production rate (i.e., after adding mean IVI, mean ITI, and mean STI to the model) and motor impairment (UPDRS-III score, with the exception of the gait domain where this score significantly contributed to the model (*B* = 0.16, *SE*(*B*) = 0.07, Wald test = 2.17, *P* = 0.03).

## Discussion

Rhythmic deficits (e.g., increased variability in rhythmic tasks) are a hallmark of PD. Here we showed that rhythmic variability is related across three motor domains—orofacial, manual (paced tapping), and gait. Rhythmic variability in the paced manual task and in the gait task, but not in the oral diadochokinesis task or in the unpaced manual task, increased with the severity in motor behavior, as measured by the motor part of the MDS-UPDRS. In general, rhythmic variability was not linked with age, disease duration, Hoehn and Yahr stage, or cognitive performance. This is in line with studies that aimed at discerning general motor performance from specific rhythmic variability. Actually, while l-dopa uptake is consistently found to reduce characteristic motor symptoms of PD (akinesia, tremor, rigidity), its beneficial effects have not been clearly established for rhythmic skills.^1^ This suggests that rhythmic variability cannot be solely explained by general motor variability. Notably, perceptual rhythmic skills (tested with the beat alignment test, BAT^9^), were a very good predictor of rhythmic variability in the three motor domains we assessed.

Thus, increased rhythmic variability in motor tasks in PD cannot be merely ascribed to a general cognitive deficit, or to any effector-specific or task-specific impairment. Our findings rather are in keeping with a generalized rhythm disorder affecting both rhythm production and perception. Yet, it is worth mentioning that correlations between variability in the manual domain and the other motor domains depended on the nature of the task (paced tapping with music vs. unpaced tapping). Musically cued tapping is likely to be more demanding than unpaced tapping in terms of cognitive or motor load, needed to align movement to the perceived beat, thus potentially making this task more sensitive to rhythmic variability. This discrepancy is not found in the other domains, though, where paced conditions are consistently found to improve rhythmic performance in axial symptoms (e.g., in gait^10^). Overall, these results, in line with previous findings,^11^ suggest that musically paced conditions may be particularly suitable for uncovering rhythmic deficits in the manual domain in PD.

Notably, relations between gait dysrhythmia (i.e., freezing episodes) and manual tapping (e.g. ref. ^7^) or between gait measures and oral rhythmic performance^8^ were previously reported in PD. These relations may result from common altered timing mechanisms subserving motor planning or initiation. Most importantly, our finding that rhythm perception predicts rhythmic variability across motor domains goes beyond the idea of an explanation purely based on the alteration of motor control.

Which mechanism is likely to underpin a general rhythm disorder in PD? A probable candidate would be a deficit of predictive timing, defined as a non-specific function involved in the ability to predict accurately the upcoming event (e.g., the next syllable, the next motor tap, or the next perceptually attended event).^12–14^ As predictive timing is neither effector-specific nor task-specific and manifests in both motor and perceptual timing tasks, it is likely to underlie global alteration of rhythmic skills in PD. The putative involvement of malfunctioning mechanisms that may serve a general predictive function in rhythm-related tasks is appealing as some causal factors have been recently identified in PD.^15^ Our findings touch upon another relevant question, namely the involvement of the basal ganglia and the cerebellum in rhythm processing. Patient and neuroimaging data indicate that both the basal ganglia and the cerebellum play a pivotal role in rhythm perception and production.^3,6^ Whilst basal ganglia are mostly involved in the internal generation of the beat,^1,3^ tapping to an external rhythm as well as detecting whether a metronome is aligned to a beat also engage the cerebellum.^3^

Indeed, speech is inherently tied to time.^16,17^ Alteration of speech pauses and pace suggest impaired speech rhythm and timing organization in PD,^4^ thus including dysarthric speech among rhythm-related symptoms in PD. Rhythmic activity, acting as an “internal model”, influences the temporal organization of speech production.^9,16^ Emerging literature suggest that rhythm metrics in speech, and particularly connected speech, might be appropriate for both diagnosis and defining outcome measures, current opinion considering that abnormalities in speech articulatory rate and regularity might represent a marker of disease progression in PD.^18^ Consequently, despite the fact that oral diadochokinesia may reflect speech—at least partially—one can imagine that poor rhythm perception might influence speech production in PD. This possibility is consistent with influential models of speech production, such as the DIVA model,^19^ implying that impaired speech rhythm in PD may partly result from perceptual rhythmic deficits. In addition, this is in agreement with a recent proposal that PD dysarthria is associated with a loss of speech motor representations, suggesting that self-monitored perceptual deficits may impoverish speech production.^20^ Further investigation is needed to shed light on the role of perceptual rhythmic deficits—as well as the contribution of cerebellum—in speech and orofacial control PD impairments. Another line of research in the non-speech literature suggests a key role of perceptual rhythmic skills in motor production, and a reciprocal influence of rhythmic movement on rhythm perception abilities.^11^ In sum, perceptuo-motor regulation loops are likely to be indiscriminately engaged in PD rhythmic skills.

These results may have clinical implications. A short and easy-to-administer rhythm perception task (e.g., BAT^9^), as opposed to a thorough clinical assessment, may be useful as a screening tool, which could inform health professionals interested in using rhythm-based interventions for alleviating both peripheral (e.g., impaired manual movement) and axial rhythm-related symptoms (e.g., impaired orofacial control, freezing of gait^21^). Furthermore, as our results—together with others^22–24^—provide evidence for an impaired central system which underpins rhythm processing, a subsequent hypothesis is that beneficial effects of a rhythmic training, which targets one specific effector, may—at least partially—transfer to other effectors. Though appealing, this hypothesis will need disentangling mechanisms that participate, for example, in speech production^25^ or freezing of gait episodes.^26^ In the latter case, we know that freezing of gait is characterized by a multifaceted pathophysiology, involving the generation and control of movement,^27^ as well as non-motor functions (e.g., executive functions^28^). Future studies will have to take into consideration these factors before examining potential transfer effects from one motor domain to the other as a result of rhythm-based interventions.

Another issue is to know whether impaired timing in PD, notably in gait or speech production, results from a primary deficit or from a compensating mechanism. Such compensation strategies to optimize gait or articulatory productions could be part of PD progression. Thus, compensatory strategies are expected to reflect cerebral pathomechanisms. For example, in the speech domain, speech production in PD is related to an altered recruitment of the principal motor regions underpinning speech production, and is associated to an increased involvement of additional areas. Changes induced by treatments mainly concern secondary motor areas and parieto-temporal regions.^29,30^ Therefore, these changes are aimed at preserving speech in PD and could reflect adjustments occurring as the disease progresses. These aspects call for deeper investigations, including in the manual and gait domains.^31,32^

Overall, the hypothesis that there may be a cross-effector beneficial transfer effect as a result of a rhythm-based intervention in one domain is intriguing, and should be tested in the future. In case of positive effects, one can imagine that innovative methods based on mobile technologies (e.g., home-based training of rhythmic skills using a dedicated app on a tablet device, e.g., refs. ^33,34^) would be a valuable complement to traditional therapeutic approaches.

In spite of these encouraging results, the study presents some limitations. The first relates to the discrepancy between the two manual rhythmic tasks (paced vs. unpaced). Indeed, mostly variability in the manual paced task showed a relation with the other rhythmic production domains (i.e., orofacial and gait). Whether this discrepancy is linked to greater variability in a paced condition compared to an uncued condition selectively in the manual domain is worth further investigation. Another limitation is inherent in the way gait measures were obtained. The instrumented gait mat used in the study afforded the recording of short gait trials (i.e., walking on an 8-m distance per trial), which were then averaged. Short trials may have put particular demands on attention, as compared to walking on a longer distance. Therefore, additional testing will be needed using a different apparatus (e.g., wireless IMU-based motion capture system) affording longer trial recordings. Finally, the study was conducted on a relatively small sample size and in the absence of a control group. The testing of a larger sample of patients, as compared to a control group of healthy older adults, will improve statistical power and allow drawing more robust conclusions.

## Methods

### Patients

Twenty-two PD patients (mean age: 69.50, SD: 5.44; 5 females; age range: 61–82), participated in the experiment (Table 1). PD was diagnosed from 4 to 25 years prior to this experiment (mean duration: 11.10, SD: 6.04) in accordance with the UK Brain Bank criteria.^35^ Each patient underwent a neurological examination performed by a neurologist specialized in movement disorders (CG) using the MDS-UPDRS scale,^36^ and a neuropsychological examination. They scored at 2-to-3 on the Hoehn and Yahr Scale, attesting a moderate PD severity. At the time of the experiment, patients were under stable dopaminergic medication since at least 4 weeks prior to the examination; they performed all evaluations and experimental tasks under optimal medication, 60–90 min after the morning dose. None of the patients experienced peak-dose dyskinesia based on clinical observation during the examination. A total score <20 on the MoCA battery,^37^ severe motor fluctuations (MDS-UPDRS 4.4 item >2), severe dystonia and dyskinesia, incapacity to walk without aid (e.g., with a stick or a walker), non-corrected auditory or visual impairment, other medical problems interfering with the proposed study, and presence of additional neurological, psychiatric, or behavioral disorders were the exclusion criteria. This study was approved by the local Ethics Committee (Comité de Protection des Personnes, Montpellier University Hospital, France; CPP No. 2015-A01090-49). All participants were recruited in the neurology ward of the Montpellier University Hospital, and participated after signing an informed consent form in accordance with the Helsinki Declaration.

### Rhythmic skill assessments

To assess rhythmic abilities across domains we used classical rhythmic tasks that were either paced (i.e., musical cueing for the manual task) or unpaced (for orofacial and gait tasks). This choice is guided by our primary aim to capture maximal rhythmic variability among PD individuals, and is informed by evidence that variability of rhythmic performance, rather than accuracy of performance, is a good indicator of rhythmic abilities.^1^ Unpaced conditions were chosen for orofacial and gait domains because (i) it has been shown that PD patients display similar regular behavior as healthy controls in a paced diadochokinetic task (e.g., in ref. ^23^), and (ii) it is well established that PD patients instantly benefit from rhythmic cueing in gait.^10^ The chosen tasks were thus expected to be highly sensitive to rhythmic disorders. For comparison, an unpaced finger tapping task was also used for testing whether variability in spontaneous manual rhythmic production was related to variability in orofacial and gait tasks.

Orofacial rhythmic abilities were tested with an oral diadochokinesis task (repetition of a pseudoword at a fast rate for 30 s),^38^ providing measures of orofacial motor control, irrespective of speech dimensions, such as intonation or phonetic components. Patients repeated the three-syllable pseudoword *pataka*; their productions were acquired with a suitable digital recorder (Zoom H4SP^©^). Audio files were further pre-processed and analyzed using Praat software.^39^ Errors, hesitations, and breathing pauses were systematically discarded for the analyses. Manual and perceptual rhythmic skills were assessed with tasks from the Battery for the Assessment of Auditory Sensorimotor and Timing Abilities (BAASTA).^40^ BAASTA is sensitive to timing and rhythm deficits in a variety of disorders including PD.^11^ In the paced manual rhythmic task, the ability to synchronize to the beat of a musical stimulus was tested. Participants were asked to synchronize their taps to the beat of a well-formed musical excerpt from Bach’s “Badinerie” and from Rossini’s “William Tell Overture” (quarter note ISI = 600 ms), each including 64 beats. The taps corresponding to the first 10 beats were systematically discarded before further analyses. The tapping trial for each musical excerpt was repeated twice. In the unpaced manual rhythmic task, participants were instructed to tap regularly at a comfortable rate for 60 s in the absence of a pacing stimulus, while maintaining tapping rate as constant as possible. In the rhythm perception task (beat alignment test—BAT^9,40^), the patients assessed whether a sequence of tones was aligned or not with the beat of short musical excerpts. BAASTA tests were administered using a tablet device (LG^©^ G Pad 8.0 model), while auditory stimuli were delivered over headphones (Sennheiser^©^ HD201). For the gait task, participants had to walk along a computerized walkway (GAITRite^©^ system) at their preferred speed for a distance of 8 m. To avoid variability (accelerations and decelerations) at the onset of the gait trial, participants started walking 2 m before the starting edge of the walkway and continued walking 2 m after the end of the walkway. Patients performed the task three times, and data were averaged.

### Variables and analyses

Participants’ rhythmic variability was assessed across the motor domains by computing the standard deviation (SD) of event intervals in the rhythmic tasks (*Orofacial variability* for inter-vowel intervals—IVIs; *Manual variability* for inter-tap intervals—ITIs; *Gait variability* for stride time intervals—STIs). The higher is the variability, the worse is the performance. Finally, for assessing perceptual rhythmic skills, a beat perception score was obtained from the BAT, the *d*′ sensitivity index, which is an unbiased measure for detecting misaligned metronome-beats. It is calculated from the number of Hits (when unaligned tones were correctly detected) and False alarms (when lack of alignment was incorrectly reported). *d*′ is the difference between the *Z*-transformed Hits rate and False Alarm rate.

To test whether variability in the three motor domains was related, we used non-parametric Spearman’s correlations, as most data were not normally distributed according to Shapiro–Wilk test. We further assessed whether demographic, clinical, and neuropsychological characteristics were related to rhythmic variables also using non-parametric Spearman’s correlations.

Finally, we tested whether rhythm perception can predict rhythmic variability in the three motor domains by using logistic regression modeling. Patients were divided into two subgroups for each motor task after a median split based on rhythmic variability (patients with less rhythmic variability vs. patients with more rhythmic variability). The two subgroups in each motor production were entered as a binary dependent variable (0: less rhythmic variability; 1: more rhythmic variability). The predictor in each model was the performance in the rhythm perception task (*d*′). The contribution of potentially confounding variables (clinical, demographic, and neuropsychological) was tested by successively entering and removing these additional predictors from the models. To control for inter-individual differences in terms of production rate, mean IVI, mean ITI, and mean STI were successively added to the models. Statistics were computed using *R* software.^41^ All significant effects were set at *P* < 0.05.

### Reporting summary

Further information on research design is available in the Nature Research Reporting Summary linked to this article.

## Supplementary information

Reporting Summary

## Data Availability

The datasets generated and/or analyzed during the current study are available from the corresponding author on reasonable request.

## Supplementary information

**Supplementary information** accompanies the paper on the *npj Parkinson’s Disease* website (10.1038/s41531-019-0092-6).
